# The Effect of Antiarrhythmic Drugs on the Beat Rate Variability of Human Embryonic and Human Induced Pluripotent Stem Cell Derived Cardiomyocytes

**DOI:** 10.1038/s41598-019-50557-7

**Published:** 2019-10-01

**Authors:** Julius Niehoff, Matthias Matzkies, Filomain Nguemo, Jürgen Hescheler, Michael Reppel

**Affiliations:** 1 Department of Diagnostic and Interventional Radiology, University Hospital of Cologne, Cologne, Germany; 2 Institute for Neurophysiology, University of Cologne, Cologne, Germany

**Keywords:** Drug safety, Heart development, Heart development

## Abstract

Embryonic stem cell (ESC) derived tissue is a promising tool to be used in different clinical, preclinical and also scientific settings, for example as *in vivo* biological pacemaker, preclinical drug safety screening tool or ultimately as part of a cell replacement therapy. However, before ESC derived tissue can be used routinely for these purposes in humans, further studies are needed. In this context, the aims of the present study were to examine the effect of antiarrhythmic drugs on human ESC (hESC) und human induced pluripotent stem cell (hiPSC) derived cardiomyocytes by analyzing the beat rate variability (BRV), which can be considered as the *in vitro* equivalent of the heart rate variability (HRV) *in vivo*. Short-term recordings of extracellular field potentials of spontaneously beating cardiomyocytes derived from hESCs and hiPSCs were made using Microelectrode Arrays (MEA). The effect of Flecainide, Ivabradine and Metoprolol was tested. The offline analysis of the BRV was mainly focused on time domain methods. Additionally a non-linear analysis method was used. The evaluation of the Poincaré-Plots of the measurements without pharmacological intervention revealed that the vast majority of the scatter plots have a similar, ellipsoid shape. Flecainide and Ivabradine influenced BRV parameters significantly, whereas Metoprolol did not alter the BRV markedly. We detected remarkable similarities between the BRV of hESC and hiPSC derived cardiomyocytes *in vitro* and the HRV *in vivo*. The effect of antiarrhythmic drugs on spontaneously beating cardiomyocytes derived from hESC and hiPSC was generally consistent with clinical experiences and also with our previous study based on murine ESC derived cardiomyocytes. In conclusion, our study points out the great potential of hESC and hiPSC derived tissue to be used routinely for many different applications in medicine and science.

## Introduction

The use of embryonic stem cell (ESC) derived tissue has been discussed in many clinical, preclinical and scientific fields. Among others, the use of this tissue as biological pacemaker and ultimately as part of a cell replacement therapy has been mentioned in the literature^1,2^. In this context, we have previously examined the effect of antiarrhythmic drugs on the beat rate variability (BRV) of murine embryonic stem cell (mESC) derived cardiomyocytes in order to demonstrate the potential of this *in vitro* tissue to serve as a preclinical drug safety screening tool^3^. However, before ESC derived tissue can be used routinely for purposes in humans, further studies are needed.

With regard to the use in clinical and preclinical settings, human embryonic stem cell (hESC) derived tissue and, in particular, human induced pluripotent stem cell (hiPSC) derived tissue is often mentioned in the literature as a valuable source^2^. In order to advance the use of ESC derived tissue, for example as part of a drug safety screening tool, the purpose of the present study was to examine the effect of antiarrhythmic drugs on hESC und hiPSC derived cardiomyocytes. It is of great importance that ESC derived tissue used for cell replacement therapies in human hearts shows similar response to cardioactive drugs as native tissue. The effect of the antiarrhythmic drugs was evaluated by analyzing the BRV, which can be considered as the *in vitro* equivalent of the heart rate variability (HRV) *in vivo*^1,3^. Mandel *et al*. have previously shown that cardiomyocytes derived from hESC and hiPSC generally exhibit BRV^1^.

HRV describes the physiological fluctuations of the time interval between successive heartbeats in humans, respectively the instantaneous heart rate^4^. In healthy individuals HRV is mainly the expression of the sustained impact of the autonomic nervous system that continuously influences the heart rate via vagal and sympathetic signals^4,5^. HRV cannot only be affected by physiological processes, such as respiration and blood pressure regulation, but also by certain cardiac and non-cardiac specific diseases, such as myocardial infarction and diabetic neuropathy^5–10^. Moreover, many clinically established antiarrhythmic drugs, such as Flecainide, Ivabradine and Metoprolol, can influence the HRV *in vivo*.

Clinical studies that examined the effect of Flecainide in patients after acute myocardial infarction and in patients without structural heart disease describe a decrease of time domain parameters of HRV^11,12^. Furthermore, case reports describe reduced fetal HRV after prenatal treatment with Flecainide^13,14^.

The administration of Ivabradine to patients suffering from non-ischemic heart failure causes an increase of HRV parameters^15^. An *in vitro* study based on spontaneously beating cardiomyocytes that were derived from hESCs and hiPSCs did not detect an effect of 10^−6^ M Ivabradine on the BRV^16^.

Depending on the patient population, the effect of β-blockers on time domain parameters of HRV is described controversially; β-blockers either increase or do not affect HRV parameters^17–20^.

In accordance with these clinical experiences, we demonstrated in a previous study that antiarrhythmic drugs influence the BRV of spontaneously beating cardiomyocytes derived from mESCs. We found great similarities between the drug effects *in vivo* and *in vitro*^3^. Ivabradine increased standard BRV parameters, whereas Flecainide significantly decreased the BRV^3^. Metoprolol did not alter time domain parameters significantly^3^.

In the present study, we made use of the Microelectrode Array (MEA) technique that allows measurements of extracellular field potentials with high temporal and spatial resolution^1,3,21,22^. This technique has previously underlined its great potential to serve as a preclinical drug safety screening tool in numerous studies^3,21–24^. The analysis of the BRV was primarily focused on time domain methods as standard analysis method of HRV in clinical settings. As a supplemental tool we created Poincaré-Plots, a non-linear analysis method.

In line with the findings of Mandel *et al*., we found that BRV was present in hESC and hiPSC derived cardiomyocytes^1^. As we have shown previously in murine tissue, the Poincaré-Plot analysis showed strong similarities between BRV *in vitro* and HRV *in vivo*. Furthermore, we found that standard antiarrhythmic drugs can influence the BRV *in vitro*.

## Material and Methods

### Cell culture and differentiation

The human embryonic stem cell line *HES-2*^25^ and the human induced pluripotent stem cell line *Foreskin C1*^26–28^ were used in this study. The cell culture of hESCs and hiPSCs, as well as the cell differentiation, followed well-established protocols that have been described in detail before^27–29^.

In short, the ESCs were co-cultured on a monolayer of inactivated fibroblast feeder cells (cell line CF1). The culture medium consisted of Dulbecco’s modified Eagle Medium (DMEM/F12 + GlutaMAX) supplemented with KnockOut Serum Replacer, non-essential amino acids, 2-Mercaptoethanol, Penicillin/Streptomycin and basic fibroblast growth factor (PeproTech Inc.). Cells were passaged once a week by manual dissection of the cell clusters.

The differentiation of hESCs and hiPSCs into embryoid bodies (EBs) was carried out in co-culture on a monolayer of feeder cells (cell line END2)^29,30^. The differentiation medium consisted of DMEM/F12 + GlutaMAX, fetal bovine serum, non-essential amino acids, 2-Mercaptoethanol and Penicillin/Streptomycin. Spontaneously beating EBs used for experiments were selected after about 4 weeks of differentiation.

The work with human ESCs has been approved by the regulatory authorities at the Robert Koch Institute, Berlin, Germany (permit number 1710-79-1-4-2). If not stated otherwise, all media and reagents were purchased from Gibco^®^ (Invitrogen, Karlsruhe, Germany).

### Experimental setup

The experimental setup has been used and described previously in several different studies^3,21,24,31^. Standard MEA chambers (60 electrodes, 30 µm electrode diameter, 200 µm inter-electrode distance) in combination with the MEA1060-Inv-BC amplifier and data acquisition system from Multichannel Systems (MCS, Reutlingen, Germany) were used to record extracellular field potentials of spontaneously beating cardiomyocytes^31^. Measurements were performed with a sampling rate of 2 kHz.

An established perfusion system based on a customized perfusor (B. Braun Melsungen AG, Germany) was used to ensure a continuous flow of 500 µl per minute and a constant concentration of the pharmacological compound within the MEA chamber^3^. A heating plate as well as a heating cannula (PH01, MCS) ensured a constant temperature of 37 °C; both were controlled automatically by a temperature controller (TC 02, MCS).

### Experimental procedure

The experimental procedure was performed as described previously^3^. First, the electrode area of the MEA chambers was coated with 0.1 % gelatine and fibronectine (both Sigma Aldrich, Germany). Then, spontaneously beating EBs were placed manually onto the electrode area and kept under incubator conditions for 48 hours in order to ensure sufficient tissue attachment onto the electrodes. The EBs were usually selected after about 4 weeks of differentiation. Prior to the start of an experiment, the differentiation medium in the MEA chambers was replaced by Iscove’s Modified Dulbecco’s Medium (IMDM) and the amount of medium was raised to 500 µl.

Experiments always started with the recording of a control measurement without pharmacological intervention. Then, measurements with one of the pharmacological substances were recorded. The concentration of the drug was increased after each recording. Experiments always ended with a recording after performing a washout. The standard recording time period for the control measurements as well as each concentration was eight minutes.

### Antiarrhythmic substances

The following antiarrhythmic substances were tested (all from Sigma Aldrich, Germany): Ivabradine hydrochloride, Flecainide acetate and (±)-Metoprolol (all dissolved in distilled water). Stock solutions were diluted in IMDM.

The I_f_ channel (HCN4), main target of Ivabradine, is expressed in early immature cardiomyocytes during embryonic development^32^. *In vitro* studies with hiPSC derived cardiomyocytes indicate that a functional sodium channel, main target of Flecainide, is expressed in stem cell derived cardiomyocytes^33^. The presence and functional integrity of the β-adrenergic system in hESC and hiPSC derived cardiomyocytes has been demonstrated recently^1^.

### BRV analysis

The BRV analysis was performed as described previously^3^. The analysis was focused on time domain methods. Briefly, the standard deviation of normal-to-normal intervals (SDNN) was considered as a parameter describing the overall variability, whereas the standard deviation of successive differences (SDSD) was rated as a parameter expressing the short-term variability^4,34^. In order to remove the influence of the beating frequency on the calculation of BRV parameters, the coefficient of variation (CV, SDNN divided by the mean normal-to-normal interval) was included in the analysis of this study^35^.

In addition, a non-linear method was used to visualize the BRV. For this reason, Poincaré-Plots that plot each time interval between two successive beats (inter-beat interval (IBI_n_)) against the following interval (IBI_n+1_) were generated for measurements without pharmacological intervention. The standard Poincaré-Plot descriptors SD1 (expressing the short-term variability) and SD2 (expressing the long-term variability) were calculated for each plot; both descriptors are highly correlated with the time domain parameters^34,36,37^.

The shape of a Poincaré-Plot displays detailed information about the variability of the heart rate *in vivo* (and the beating rate *in vitro)*, thus allowing conclusions about the health status of a patient from a clinical perspective. Typically Poincaré-Plots have a cigar-, ball- or fan-shape^37,38^. We have created Poincaré-Plots for all measurements without pharmacological intervention of each individual EB and visually categorized the Poincaré-Plot according to the shape of the plot.

### Data analysis

A customized macro script for the Spike2-software (Cambridge Electronic Design, Cambridge, UK) was used for the analysis of the raw MEA data and the calculation of the BRV parameters^3^.

In order to ensure the highest possible comparability with our previous study based on mESC derived tissue, we applied the same exclusion criteria for the elimination of artifacts and outliers^3^:

- The recording of a single concentration of a measurement that differed from the corresponding control measurement in terms of recording time was excluded (as reasoned in^4^).
- A complete measurement was excluded in case the values for SDNN and SDSD of the lowest applied concentration were < 60 % of the corresponding control measurement.
- The recording of a single concentration of an experiment was excluded in case the value of one of the observed parameters differed by more than two standard deviations from the mean of all measurements with this particular concentration.
- In the case of three or more concentrations of the same experiment meeting criterion (c), the complete measurement was excluded.

The results are displayed as mean ± standard error of the mean (SEM). Statistical analysis was performed by the Wilcoxon-Test (every concentration compared to control) using SPSS software (IBM, USA). P-values ≤ 0.05 were considered statistically significant.

### Ethical approval

The work with human ESCs has been approved by the regulatory authorities at the Robert Koch Institute, Berlin, Germany (permit number 1710-79-1-4-2). The experiments were carried out in accordance with the relevant guidelines and regulations.

## Results

The analysis of the BRV *in vitro* (without pharmacological intervention) was based on the measurements with a total of 21 beating clusters (EBs) derived from hESCs and 19 beating clusters (EBs) derived from hiPSCs. The mean beating frequency as well as the mean SDNN, SDSD and CV are presented in Table 1.

**Table 1:** Results for control measurements without pharmacological intervention.

Cell Type	Total Number of EBs	Mean Beating Frequency	Mean SDNN	Mean SDSD	Mean CV
hESC	21	1.30 ± 0.07 Hz (range 0.74–1.85 Hz)	17.21 ± 1.84 ms	6.03 ± 0.93 ms	0.020 ± 0.002
hiPSC	19	1.26 ± 0.07 Hz (range 0.78–1.92 Hz)	26.37 ± 4.32 ms	11.32 ± 2.92 ms	0.030 ± 0.003

Data is presented as mean ± SEM.

### Poincaré-Plot analysis

**Figure 1:**
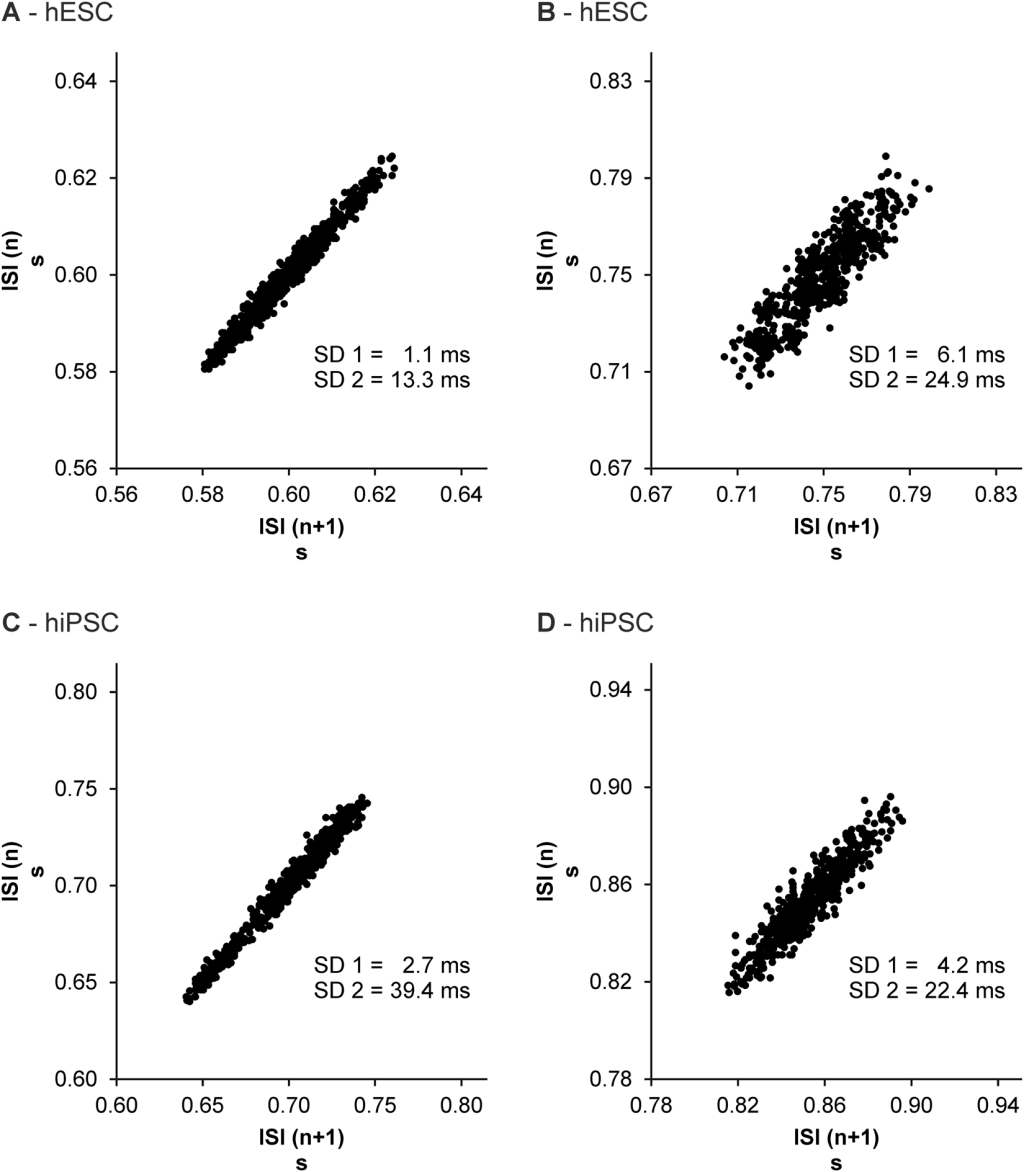
Poincaré-Plots demonstrating the BRV of hESC derived (**A,B**) and hiPSC derived (**C,D**) cardiomyocytes. Most scatterplots generated for measurements without pharmacological intervention had an ellipsoid shape. ISI = inter-beat interval.

The evaluation of the Poincaré-Plots generated for measurements without pharmacological intervention revealed that the vast majority of EBs derived from hESCs as well as hiPSCs exhibit a similar shape of scatterplot (Fig. 1). With the exception of very few measurements, most scatterplots had an ellipsoid shape; partly with a small minor axis (see Fig. 1A,C), partly with a wider minor axis (see Fig. 1B,D), which is also expressed by the values of SD1.

### Flecainide

Flecainide was applied in a concentration range from 10^−10^ M to 10^−5^ M. In clinical trials the peak plasma concentration (C_max_) of Flecainide was approximately 4.1 × 10^−6^ mol/L after intravenous administration^39^.

#### hESC

**Figure 2:**
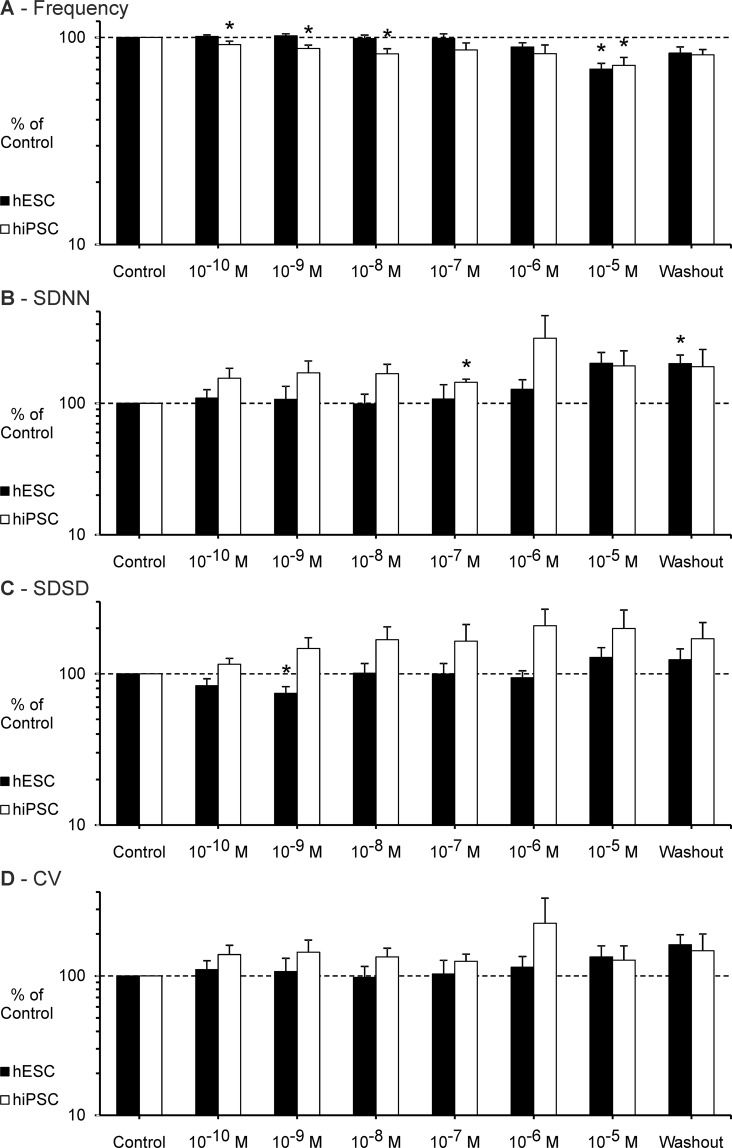
Effect of Flecainide on the BRV of hESC and hiPSC. (**A–D**) Beating frequency, SDNN, SDSD and CV figured as mean ± SEM. Flecainide decreased the beating frequency and increased the BRV parameters. Note the significantly reduced short-term variability (SDSD) at a concentration of 10^−9^ M (hESC). Measurements performed on seven hESC derived EBs (n = 7) and six hiPSC derived EBs (n = 6) were included in the analysis. Each EB was measured once. *p ≤ 0.05.

Experiments with seven different EBs were included in the analysis of the effect of Flecainide on BRV parameters (see Fig. 2). After the application of the maximum concentration (10^−5^ M), the experiment with one EB had to be stopped before the full recording time of eight minutes due to markedly reduced frequency and FP amplitude; the EB recovered in terms of beating frequency and overall variability after the washout.

The beating frequency was significantly reduced at a concentration of 10^−5^ M (70.4 % ± 4.6 SEM of control, p ≤ 0.05). The short-term variability (SDSD) was significantly decreased at a concentration of 10^−9^ M (74.3 % ± 7.8 SEM, p ≤ 0.05). At higher concentrations particularly the overall variability (SDNN) was increased (201.6 % ± 41.9 SEM of control at 10^−5^ M).

Interestingly there was a diverse effect on variability parameters at a concentration of 10^−5^ M: Four EBs showed increased BRV (SDNN and SDSD), whereas two EBs showed decreased BRV.

#### hiPSC

Experiments with six different EBs were included in the analysis of the effect of Flecainide on BRV parameters (see Fig. 2). With increasing concentration of Flecainide the beating frequency of the EBs steadily decreased (73.4 % ± 6.6 SEM of control at 10^−5^ M, p ≤ 0.05) and BRV parameters increased. At a concentration of 10^−6^ M BRV parameters (SDNN and SDSD) were markedly increased in three EBs; two other EBs showed markedly decreased BRV (SDNN and SDSD). Likewise, the BRV was markedly increased in four EBs at a concentration of 10^−5^ M and at the same time reduced in two other EBs.

### Ivabradine

Ivabradine was applied in a concentration range from 10^−10^ M to 10^−4^ M. In clinical trials the peak plasma concentration (C_max_) of Ivabradine was approximately 2.6 × 10^−7^ mol/L after intravenous administration^40^.

#### hESC

**Figure 3:**
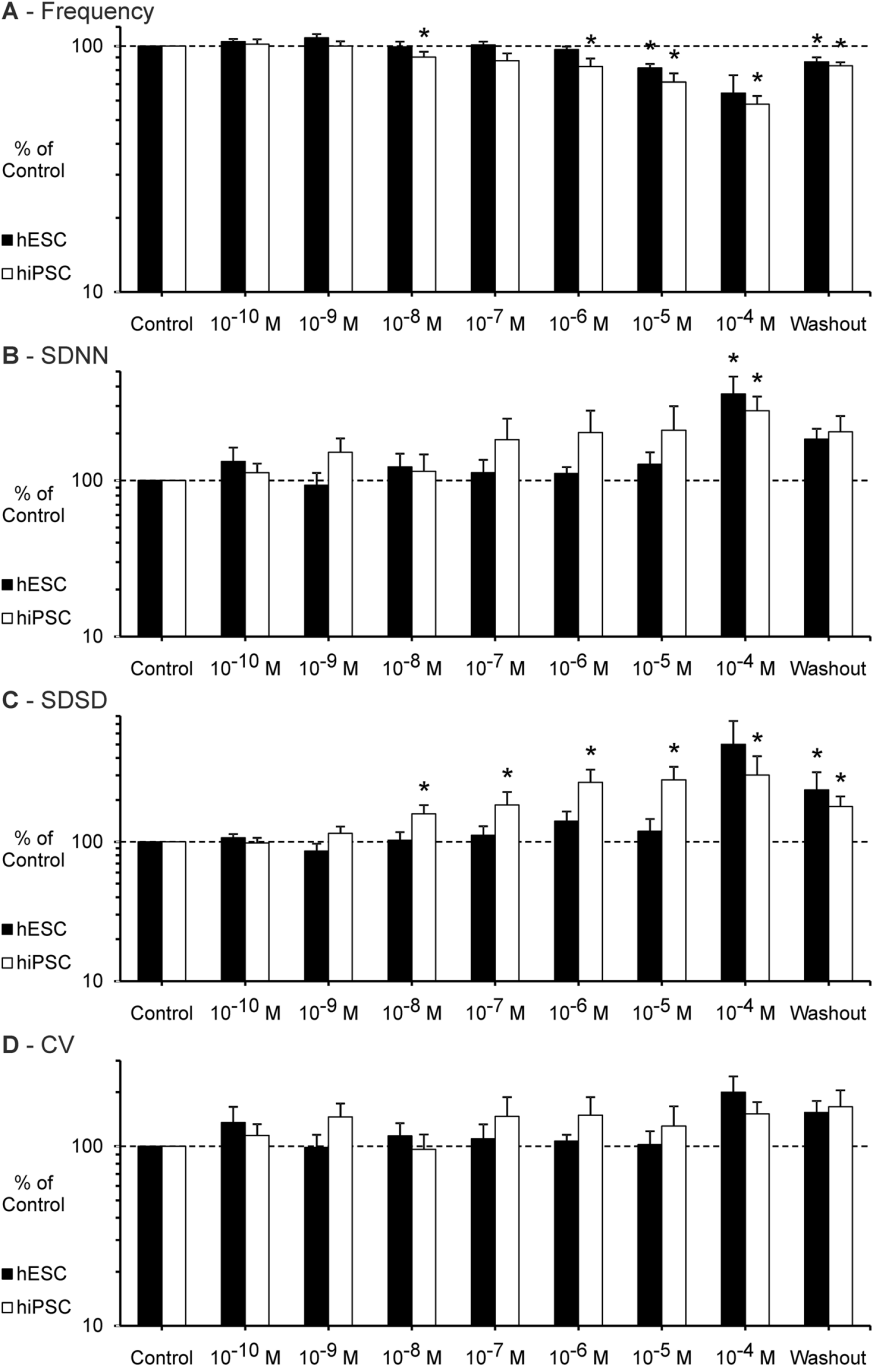
Effect of Ivabradine on the BRV of hESC and hiPSC. (**A**–**D**) Beating frequency, SDNN, SDSD and CV figured as mean ± SEM. Ivabradine decreased the beating frequency and increased BRV parameters dose-dependently. The results presented for 10^−4^ M include measurements that had to be stopped before the full recording time of eight minutes. Measurements performed on seven hESC derived EBs (n = 7) and six hiPSC derived EBs (n = 6) were included in the analysis. Each EB was measured once. *p ≤ 0.05.

Experiments with seven different EBs were included in the analysis of the effect of Ivabradine on BRV parameters (see Fig. 3). After the application of the maximum concentration (10^−4^ M), the experiments with three EBs had to be stopped before the full recording time of eight minutes due to markedly reduced frequency and FP amplitude; the EBs recovered after the washout.

Ivabradine caused a markedly decrease of beating frequency (64.4 % ± 11.8 SEM of control at 10^−4^ M) and an increase of overall variability as well as short-term variability up to a maximum level at 10^−4^ M (SDNN 357.9 % ± 104.4 SEM of control, p ≤ 0.05; SDSD 502.1 % ± 234.6 SEM of control).

#### hiPSC

Experiments with six different EBs were included in the analysis of the effect of Ivabradine on BRV parameters (see Fig. 3). After the application of the maximum concentration (10^−4^ M), the experiments with four EBs had to be stopped before the full recording time of eight minutes; two out of four EBs recovered in terms of frequency and variability after the washout, the other two EBs showed partial recovery after the washout.

As the concentration of Ivabradine increased, the beating frequency of the EBs steadily decreased (58.1 % ± 4.6 SEM of control at 10^−4^ M, p ≤ 0.05). At the same time the variability parameters steadily increased to a maximum level at 10^−4^ M (SDNN 279.7 % ± 65.2 SEM of control, p ≤ 0.05; SDSD 301.7 % ± 110.1 SEM of control, p ≤ 0.05).

### Metoprolol

Metoprolol was applied in a concentration range from 10^−10^ M to 10^−4^ M. In clinical trials the peak plasma concentration (C_max_) of Metoprolol was approximately 8.0 × 10^−7^ mol/L after oral administration^41^.

#### hESC (data not shown)

Experiments with seven different EBs were included in the analysis of the effect of Metoprolol on BRV parameters. The greatest impact on the beating frequency was observed after the application of 10^−4^ M Metoprolol (84.6 % ± 6.7 SEM of control). Neither the overall variability (SDNN) nor the short-term variability (SDSD) was affected significantly by the β-blocker.

#### hiPSC (data not shown)

Experiments with seven different EBs were included in the analysis of the effect of Metoprolol on BRV parameters. Neither the beating frequency nor any of the observed BRV parameters was affected significantly by Metoprolol.

## Discussion

In the past decade, scientists have made great progress exploring the potential of ESC derived tissue and cardiomyocytes in particular. Preclinical drug safety screening is probably the most frequently mentioned application area that may be substantially improved by the use of ESC based *in vitro* models. In this context several recent studies focus on drug induced proarrhythmicity of ESC derived cardiomyocytes.

Blinova *et al*. evaluated an *in vitro* model based on hiPSC derived cardiomyocytes for studies on drug induced proarrhythmicity. Their work focused on the drug induced risk of Torsade de Pointes (TdP)^42^. Metoprolol, considered to be a low-risk drug regarding the induction of TdP, induced arrhythmia-like events *in vitro*^42^. However, the authors limit that the effects of Metoprolol may not be well displayed in their *in vitro* model because of the lack of innervation of the cardiomyocytes in monocultures^42^.

Ando *et al*. also focused their study on drug induced torsadogenic risk assessment using an *in vitro* model based on hiPSC derived cardiomyocytes^43^. Among others, Flecainide and Metoprolol were evaluated. Flecainide induced early afterdepolarization, decreased the amplitude of the 1st peak in a concentration-dependent manner and caused a stop of beating^43^. Metoprolol neither altered the field potential duration (FDP), induced early afterdepolarization, nor caused a stop of beating^43^.

Compared to other drugs Ivabradine is used less frequently in *in vitro* studies focusing on drug induced proarrhythmicity. Nevertheless, the effect of Ivabradine, known to block I_f_ channels in the sinus node, is of great interest when considering ESC derived cardiomyocytes as biological pacemakers. Chauveau *et al*. describe an attenuation of automaticity of iPSC derived cardiomyocytes by Ivabradine in a concentration-dependent manner until complete block at 10 μmol/L^44^.

The purpose of the present study was to examine the effect of antiarrhythmic drugs on hESC and hiPSC derived cardiomyocytes by analyzing the BRV. It is the first study focusing on all aspects of BRV *in vitro*. We found that the BRV of ESC derived cardiomyocytes *in vitro* shows similar characteristics of the HRV of the human heart *in vivo*. In addition, we found that Flecainide and Ivabradine can alter the BRV significantly, whereas Metoprolol has no major influence on BRV parameters.

Similarities between the effects of antiarrhythmic drugs on the BRV *in vitro* and the HRV *in vivo* are not only a strong indication for functional integrity, but also basis for drug safety screening tools and prerequisite for cardiomyoplasty. Our findings complement the recently published studies and once again emphasize the great potential of ESC based *in vitro* models.

### Poincaré-Plots

The vast majority of Poincaré-Plots generated for hESC as well as hiPSC derived cardiomyocytes had an ellipsoid shape (also described as “cigar shape” in the literature)^37,45,46^. This is a typical shape of Poincaré-Plot described for the HRV of patients in clinical studies. Also we have seen this shape in the analysis of the BRV of mESC derived cardiomyocytes^3^. Interestingly, other shapes of scatterplots (e.g. ball- and fan-shaped plots) were not as frequent as in our study based on mESC derived cardiomyocytes^3^. Nevertheless, considering the fact that Poincaré-Plots display detailed information about the variability of the heart rate *in vivo* and the beating rate *in vitro*, this non-linear analysis method could possibly point to similarities between HRV and BRV and therefore be suitable for a comparison of the variability *in vivo* and *in vitro*.

### Flecainide

The effect of Flecainide on BRV parameters of cardiomyocytes derived from hESCs is principally consistent with clinical observations and experiences obtained from *in vitro* studies^3,11,12^. Especially interesting is the finding that the short-term variability (SDSD) was significantly decreased at a concentration of 10^−9^ M; the exact same concentration that induced a decreased short-term variability in mESC derived cardiomyocytes^3^.

Reduced beating frequency and increased BRV of hiPSC derived cardiomyocytes in response to Flecainide is consistent with observations made with hESC and mESC derived cardiomyocytes^3^. In contrast to previous *in vitro* and clinical studies, there was no significant reduction of BRV parameters evoked by the application of Flecainide.

The diverse effect of high concentrations of Flecainide (increased BRV in some EBs, decreased BRV in other EBs) that was observed in hESC as well as in hiPSC derived EBs, has been observed previously in cardiomyocytes derived from mESCs^3^. Since this observation was made using cardiomyocytes derived from three different cell lines, differing control measurements of the EBs seem unlikely to be the reason for this observation. It can be speculated that pharmacodynamic variability that has been described in clinical studies and/or unspecific side effects on different ion channels might be an explanation for this diverse effect of high concentrations of Flecainide^47^. Structural differences within the EBs could be another reason for this finding.

As a class I_c_ antiarrhythmic agent, Flecainide is known to block sodium channels in cardiomyocytes^48^. Flecainide is also known to inhibit ryanodine receptors, which are major regulators of the sarcoplasmic calcium release^49^. Ben-Ari *et al*. have previously identified intracellular calcium currents, including those associated with the sarcoplasmic reticulum, as an important component in the development of the BRV^16^. As Flecainide does not selectively inhibit a single type of ion channel in cardiomyocytes, it remains unclear whether the observed drug effect is actually caused by blocking sodium channels or by influencing intracellular calcium pathways.

Nevertheless, the decreasing effect of Flecainide on BRV parameters as observed in cardiomyocytes derived from hESC and mESC is a remarkable finding that should be the subject of further studies.

### Ivabradine

In line with clinical observations and *in vitro* studies based on mESC derived cardiomyocytes Ivabradine steadily decreased the beating rate of hiPSC and hESC derived cardiomyocytes with increasing concentration^3,15,16^. Likewise, the increasing effect of Ivabradine on BRV parameters that has been reported for mESC derived cardiomyocytes could be reproduced^3^. The increasing effect on BRV parameters is also consistent with clinical observations^15^.

In accordance with the study of Ben-Ari *et al*., there was no significant effect of 10^−6^ M Ivabradine on the BRV (SDNN)^16^. Therefore, it can be concluded that an acute blockade of the I_f_ currents with low concentrations of Ivabradine has no major influence on the BRV of hESC and hiPSC derived cardiomyocytes *in vitro*. However, higher concentrations of Ivabradine can cause a significant increase of BRV parameters – just as it is known from clinical trials^15^.

The pharmacological properties of the drug may possibly provide an explanatory approach for these findings. Ivabradine is known to bind specifically to the *funny*-channel at therapeutic concentrations^50^. However, it is also possible that Ivabradine operates less specifically at higher concentrations^50,51^. Thus, it remains unclear whether the effect on BRV parameters caused by high concentrations of Ivabradine is actually due to a specific blockage of the I_f_ currents or whether an unspecific blockade of different ion channels contributes to the effect.

Furthermore, it has to be noted that the findings in clinical trials are from patients with a cardiac disease. It is unknown whether these cardiac diseases are associated with changes in intracellular signaling pathways that might have an additional influence on the drug effect.

### Metoprolol

Consistent with the effect on mESC derived cardiomyocytes, Metoprolol neither altered the overall (SDNN) nor the short-term variability (SDSD) of hESC and hiPSC derived cardiomyocytes markedly.

Also consistent with previous studies is the finding that β-blockers do not exhibit a great impact on the beating rate of ESC derived cardiomyocytes *in vitro*^3,52^.

Metoprolol is a selective β_1_-receptor antagonist^53^. In general, β-receptors are a common receptor type in humans and essentially involved in many physiological processes associated with the β-adrenergic system.

It has previously been demonstrated that β-blockers are able to antagonize the chronotropic effect of catecholamines on ESC derived cardiomyocytes *in vitro*^1^. However, it is interesting that the sole application of Metoprolol has no major influence – neither on the beat rate nor on the BRV. These findings are also supported by other *in vitro* studies^3,52^. This observation could lead to the assumption that the β-adrenergic system does not play a decisive role in the physiology of the ESC derived cardiomyocytes within the EBs.

Before drawing any conclusions about the human embryonic phase from these findings, it must be remembered that the physiological processes within an EB have not yet been fully discovered and therefore might differ from those of a human embryo.

### Comparison of different cell lines

In general, the experiences made with hESC and hiPSC derived EBs were comparable – not only regarding the handling of the cell cultures and the cell differentiation, but also regarding the response to cardioactive drugs. The minor differences between these human cell lines cannot be fully explained due to the present study design. These differences may not be present when testing a large number of EBs.

The effects of antiarrhythmic drugs on mESC derived cardiomyocytes and on hESC and hiPSC derived cardiomyocytes show clear similarities. The diverse effect of high concentrations of Flecainide, which was observed in all three cell lines, appears particularly interesting. Also the lowering effect of Flecainide on BRV parameters observed in hESC and mESC derived cardiomyocytes is remarkable. Nevertheless, a detailed comparison between murine and human ESC derived tissue is hampered by the fact that murine embryos develop much faster than human embryos. It is most likely that the development at the cellular level and consequently also the ion channel expression in the cell membrane does not completely match between murine and human ESC derived cardiomyocytes, which influences drug effects.

Since the effects were generally comparable, the mESCs might be preferred for *in vitro* studies because of the easier handling and faster differentiation of the cells. However, if clinical use is desired, human ESC derived tissue, especially hiPSC derived tissue, should be used in addition.

## Conclusions and Limitations

The key finding of the present study is the proof that antiarrhythmic drugs can influence the BRV of spontaneously beating cardiomyocytes derived from hESC and hiPSC. In general, the effect of the tested antiarrhythmic agents was comparable to the experiences made in previous *in vivo* and *in vitro* studies.

The minor differences between the effect of antiarrhythmic drugs on murine and human ESC derived cardiomyocytes can possibly be explained by diverse ion channel expression on the cell membrane due to different developmental stages. Also, structural differences within the EBs, such as cell-cell interactions or the expression of catecholamine producing cells, may be another explanation for different drug effects.

Further studies are needed to address these issues and variables before ESC derived tissue can be used routinely as a preclinical drug testing tool and ultimately as part of a cell replacement therapy.

## Data Availability

The datasets analyzed during the current study are available from the corresponding author on request.
